# E. fischeriana Root Compound Dpo Activates Antiviral Innate Immunity

**DOI:** 10.3389/fcimb.2017.00456

**Published:** 2017-10-26

**Authors:** Jingxuan Chen, Hongqiang Du, Shuang Cui, Tong Liu, Guang Yang, Huaping Sun, Weiwei Tao, Baoping Jiang, Li Yu, Fuping You

**Affiliations:** ^1^Department of Immunology, Institute of Systems Biomedicine, School of Basic Medical Sciences, Peking University Health Science Center, Beijing, China; ^2^Key Laboratory for Neuroscience, Neuroscience Research Institute, Peking University Beijing, National Health and Family Planning Commission of the People's Republic of China (NHFPC), Ministry of Education, Beijing, China; ^3^College of Animal Science and Veterinary Medicine, Heilongjiang Bayi Agricultural University, Daqing, China; ^4^Department of Parasitology, Department of Public Health and Preventive Medicine, School of Basic Medical Sciences, Jinan University, Guangzhou, China; ^5^Jiangsu Hospital of Traditional Chinese Medicine, Affiliated Hospital of Nanjing University of Chinese Medicine, Nanjing, China; ^6^Center for Translational Systems Biology and Neuroscience, School of Basic Biomedical Science, Nanjing University of Chinese Medicine, Nanjing, China; ^7^Jiangsu Key Laboratory for High Technology Research of TCM Formulae, Jiangsu Collaborative Innovation Center of Chinese Medicinal Resources Industrialization, Pharmacy College, Nanjing University of Chinese Medicine, Nanjing, China

**Keywords:** innate immunity, viruses, RLR signalling, STING, compound

## Abstract

E. fischeriana has long been used as a traditional Chinese medicine. Recent studies reported that some compounds of E. fischeriana exhibited antimicrobial and immune enhance activity. Innate immune system is essential for the immune surveillance of inner and outer threats, initial host defense responses and immune modulation. The role of natural drug compounds, including E. fischeriana, in innate immune regulation is largely unknown. Here we demonstrated that E. fischeriana compound Dpo is involved in antiviral signaling. The genome wide RNA-seq analysis revealed that the induction of ISGs by viral infection could be synergized by Dpo. Consistently, Dpo enhanced the antiviral immune responses and protected the mice from death during viral infection. Dpo however was not able to rescue STING deficient mice lethality caused by HSV-1 infection. The enhancement of ISG15 by Dpo was also impaired in STING, IRF3, IRF7, or ELF4 deficient cells, demonstrating that Dpo activates innate immune responses in a STING/IRFs/ELF4 dependent way. The STING/IRFs/ELF4 axis is therefore important for Dpo induced ISGs expression, and can be used by host to counteract infection.

## Introduction

Virus infection poses serious threats to public health. Innate immunity is the sentinel for the host health and is promptly activated after infection. The pattern recognition receptors (PRRs) are the first line of innate immunity to sense the invading pathogens and other danger signals. Antiviral immune responses are triggered after viral recognition by host PRRs, including several Toll-like receptors (TLR3, TLR7/8, TLR9; Schulz et al., 2005; Hoshino et al., 2006), RIG-I like receptors (RIG-I and Mda5; Yoneyama et al., 2004, 2005), and cytosolic double strand DNA (dsDNA) receptors (DAI, IFI16, DDX41, and cGAS; Takaoka et al., 2007; Unterholzner et al., 2010; Zhang et al., 2011; Sun et al., 2013). RIG-I like receptors recognize cytoplasmic viral RNA, and recruit the adaptor MAVS (Mitochondrial Antiviral Signaling Protein; Seth et al., 2005; Xu et al., 2005; Kumar et al., 2006; Liu et al., 2015). MAVS localizes on mitochondria and peroxisomes, and after receiving danger signals from RLRs, recruits downstream molecules to form a signal complex. This signal complex then triggers the activation of signaling events, leading to the transcription of NFκB and IRF3/7-dependent genes. STING (Stimulator of Interferon Genes Protein, also named ERIS or MITA) was initially identified as an adaptor protein downstream of cytosolic DNA sensing pathway (Ishikawa and Barber, 2008; Zhong et al., 2008; Sun et al., 2009). The recent studies showed that STING was able to recognize the secondary message cGAMP (Cyclic GMP-AMP) synthetized by cGAS (Sun et al., 2013). After binding with cGAMP, STING recruited TBK1 to form a signal complex, and initiated the transcription of type I IFNs and related antiviral genes in an IRF3/7-dependent way.

The Euphorbia is the largest genus (Li et al., 2009) of Euphorbiaceae, which is one of the largest families of higher plants. The species are distributed worldwide (Vasas and Hohmann, 2014) throughout tropical and temperate regions, mainly growing in southern USA, the Mediterranean basin, the Middle East, South Africa and north China. Many of them have been used as folk medicine with a long history (Wang et al., 2006; Ghanadian et al., 2013). For their extensive biological activities, Euphorbia species have been used for treatment of yellow fever (Korin et al., 2002), viral hepatitis (Hezareh, 2005), edema, ascites, warts (Wang et al., 2006), cancer (Wang et al., 2009), multi-drug resistance (Wesolowska et al., 2007), skin diseases, gonorrhea, and migraine (Shi et al., 2008). Some studies focused on their antiviral activities. Abdelgaleil et al. (2001) described the compounds from *Euphorbia paralias* L. (growing in Egypt and entire Mediterranean region), which inhibited HIV (human immunodeficiency virus) induced cytopathicity in MT-4 cells. Akihisa et al. (2002) and Tanaka et al. (2000) used Raji cells, the Epstein-Barrvirus (EBV) genome-carrying human lymphoblastoid cells to assay the compounds from *Euphorbia antiquorum* L. and *Euphorbia chamaesyce* L. Seven and one of them showed inhibitory effects respectively on EBV early antigen (EBV-EA), induced by the tumor promoter 12-O-tetradecanoylphorbol-13-acetate (TPA).

Euphorbia fischeriana Steud is a perennial herbaceous plant, which produces a milky juice. The roots of E. fischeriana had long been used as a traditional Chinese medicine for the treatment of cancer, edema, and ascites. Accumulating studies are uncovering its role in antiviral immune responses. A phorbol ester from Euphorbia species (Sun and Liu, 2011), prostratin, is well-known for the notable induction activity on viral reservoirs. There are more potent analogs of prostratin, in which phorbol-13-stearate showed at least 10-fold more potent than prostratin on the activation of HIV-1 gene (Márquez et al., 2008). Besides prostratin, the role of other compounds of E. fischeriana in antiviral immunity awaited to be brought to light. The association between natural drug and innate immunity also is an interesting topic to be explored.

In present study, we demonstrated that a compound, named Dpo, which was isolated from Euphorbia fischeriana Steud, was able to activate the antiviral immunity in type I IFNs independent manner. Dpo up-regulated a group of ISGs (interferon stimulated gene) and inflammatory genes but type I IFNs. Dpo protected wild type mice from lethality due to virus infection but not STING deficient mice. We further found Dpo exerted its antiviral function through STING and IRFs. Therefore, we here revealed a new antiviral pathway triggered by Dpo.

## Result

### Dpo is an antiviral compound from euphorbia fischeriana steud

**Figure 1:**
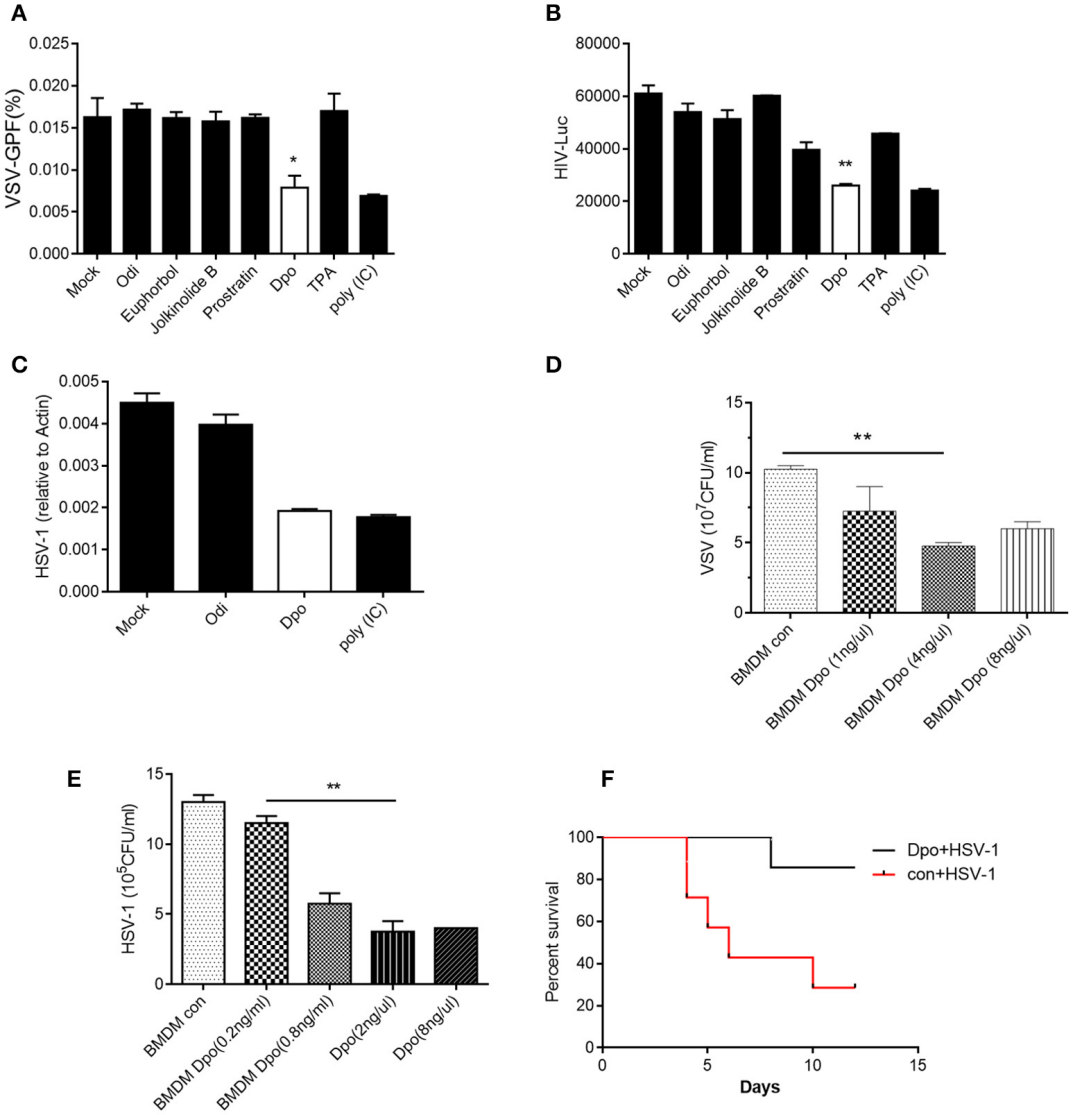
Dpo is an antiviral compound from Euphorbia fischeriana Steud. **(A–C)** Bone marrow derived macrophages were treated with indicated compounds (1 ng/ul). Four hours later, these cells were infected with VSV **(A)**, HIV-1 **(B)**, and HSV-1 **(C)**. Twenty-four hours later, the viral load were measured by plaque assay. **(D,E)** Bone marrow derived macrophages were treated with increasing amount of Dpo. Four hours later, these cells were infected with VSV **(D)** and HSV-1 **(E)**. Twenty-four hours later, the viral load were measured by plaque assay. **(F)** Wild type C57BL/6 mice were treated with Dpo, 24 h later infected with HSV-1, and monitored daily for 15 days. ^*^P < 0.05. **(A–E)** The data represent mean values ± SEM (*n* = 3); ^*^P < 0.05, ^**^P < 0.01, significant compared to control, Student's *t*-test. The data represent mean values ± SEM (*n* = 6 mice per group); **(F)** ^*^P < 0.05, significance compared to the control group, non-parametric Mann–Whitney analysis.

To investigate the function of E. fischeriana in host defense, we isolated a list of chemical compounds from it and examined their antiviral activity. Dpo is the only compound that was able to suppress the infection of VSV, a negative-sense single strand RNA virus (Figure 1A and Supplementary Figure 1). However, the replication of VSV was not affected by the treatment of prostratin, which is known as a HIV-1 inhibitor. This indicated that Dpo employed a different mechanism to restrict virus infection. To gain additional insight of the function of Dpo, we infected wild type bone marrow derived macrophages with VSV-G-HIV-1 and HSV-1 (Herpes simplex virus 1), a double strands DNA virus after Dpo treatment (Figures 1B,C). Viral replication was also inhibited by Dpo but not by other compounds. We further observed that Dpo restricted virus in a dose dependent manner (Figures 1D,E). Thus, Dpo suppressed the infection of different types of viruses significantly, suggesting that Dpo is a general anti-viral compound without virus specificity. To further evaluate the physiological function of Dpo, we challenged the wild type mice with HSV-1. The control group mice were much more susceptible to HSV-1 than Dpo treated group (Figure 1F). Therefore, Dpo is an active anti-viral compound from E. fischeriana.

### Dpo induces expression of interferon-stimulated genes

**Figure 2:**
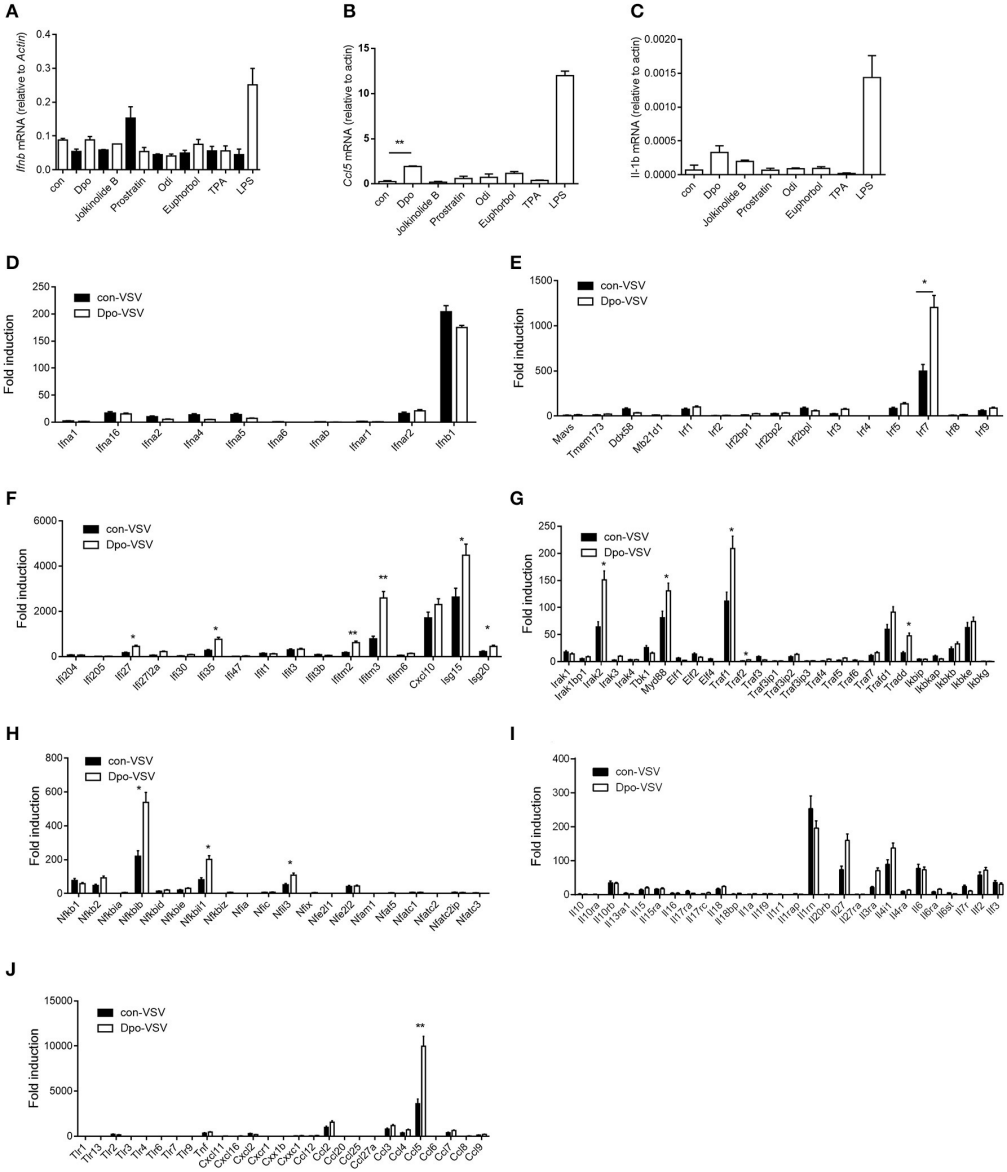
Dpo induces expression of interferon-stimulated genes. **(A–C)** Bone marrow derived macrophages were treated with indicated compounds (1 ng/ul). Four hours later, the mRNA level of IFNβ, CCl5, and IL-β were measured by qPCR. **(D–J)** Bone marrow derived macrophages were treated with Dpo. Four hours later, these cells were infected with VSV. Twenty-four hours later, the indicated cytokines and genes were measured by qPCR. **(A–J)** The data represent mean values ± SEM (*n* = 3); ^*^P < 0.05, ^**^P < 0.01, significant compared to control, Student's *t*-test.

Type I IFN is one of the most potent defensive element against virus infection. IFNβ is the earliest induced IFN during viral infection. Dpo however did not affect the production of IFNβ (Figure 2A). Interestingly CCL5 and IL1-β were up-regulated slightly after Dpo treatment (Figures 2B,C), indicating that Dpo might exert its anti-viral function in a chemokines and inflammatory cytokines dependent way. To gain further insight of the mechanism of how Dpo direct antiviral immune responses, we performed a genome wide RNA-seq to identify Dpo regulated signaling and genes. Interestingly Dpo treatment led to significant alteration of antiviral pathways in bone marrow derived macrophages, including TLR, RLR, NLR, and cytosolic DNA sensing pathway. Dpo also induced or suppressed a group of genes associated with Hepatitis A Virus, Hepatitis B Virus, Hepatitis C Virus, influenza virus, HSV and Epstein-barr virus (Supplementary Figure 2). This further suggested that Dpo suppressed virus via regulating innate immune signaling. In addition to IFNβ, IFNαs are the main effector to eliminate infected virus. Similar to IFNβ, Dpo did not induce IFNαs production in macrophages (Figure 2D). We next measured the expression of the known innate immune signaling molecules after Dpo treatment. Dpo significantly enhanced the induction of IRF7 during viral infection (Figure 2E). As IRF7 is an interferon stimulated genes (ISG). We then reason that Dpo might be involved in ISG regulation. A list of ISGs were induced by viral infection, and further enhanced by Dpo (Figure 2F). NFκB is a key transcription factor that plays a pivotal role in innate immune signaling, including the defense against virus. The RNA-seq analysis showed that Dpo regulated NFκB pathway. The q-PCR results further verified that Dpo up-regulated the adaptor proteins, including: IRAK2, TRADD, TRAF1, and MyD88 (Figure 2G), and transcription factors, including: NFκB2, NFκBIB, NFκBil1, and NFil3 (Figure 2H). Accordingly, Dpo enhanced the expression of many inflammatory cytokines (Figure 2I) and chemokine during virus infection (Figure 2J). Therefore, Dpo enhanced virus induced immune genes expression, particularly the ISGs induction, which is critical for its anti-viral function.

### Sting is required for Dpo mediated anti-viral immune responses

**Figure 3:**
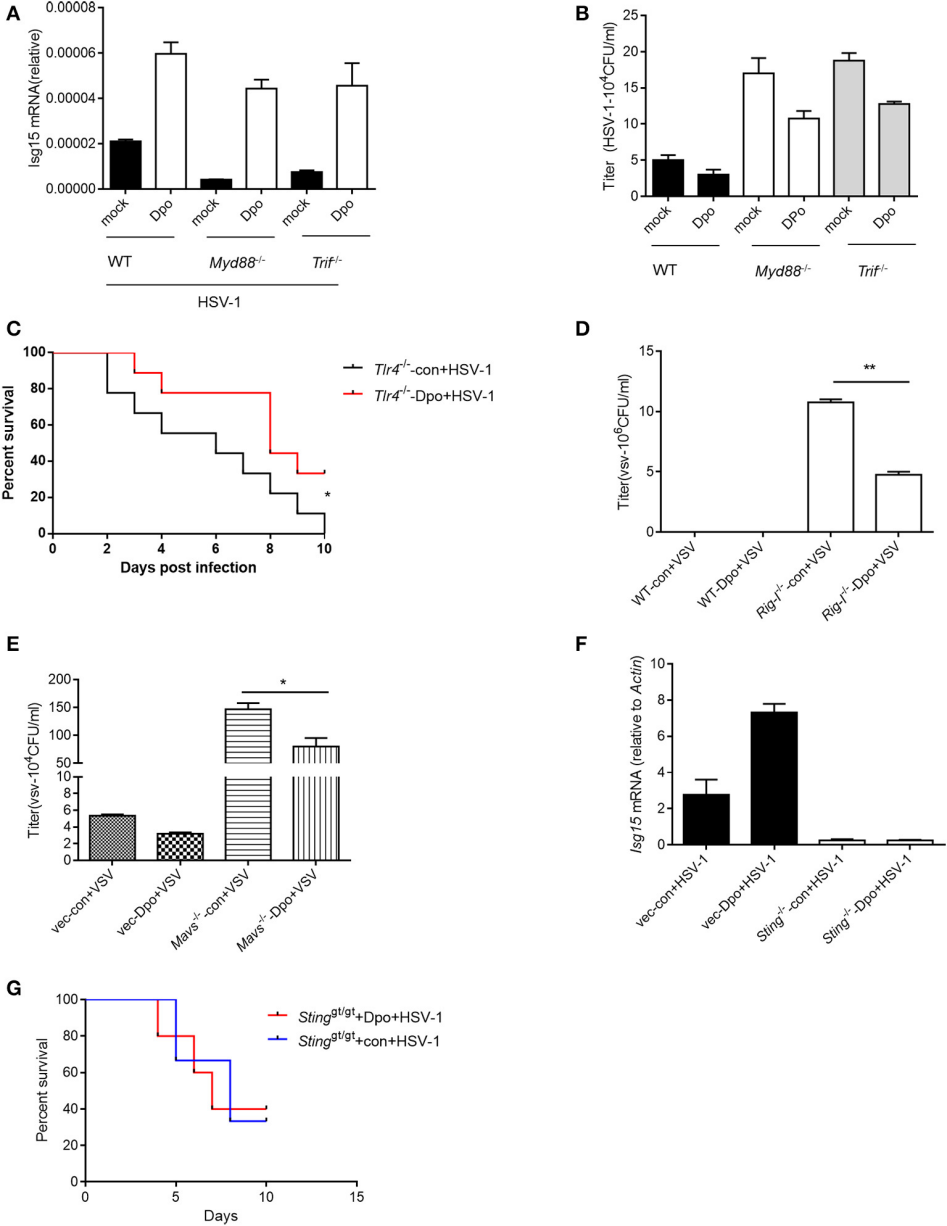
STING is required for Dpo mediated anti-viral immune responses. **(A,B)** WT, *Trif* ^−/−^, *Myd88*^−/−^ peritoneal macrophages were treated with Dpo. Four hours later, these cells were infected with HSV-1. Twenty-four hours later, the mRNA level of ISG15 were measured by qPCR **(A)**, and the viral load were measured by plaque assay **(B)**. **(C)** *Tlr4*^−/−^ C57BL/6 mice were treated with Dpo, 24 h later infected with HSV-1, and monitored daily for 15 days. **(D,E)** WT, *Ddx58*^−/−^ **(D)**, *Mavs*^−/−^ **(E)** peritoneal macrophages were treated with Dpo. Four hours later, these cells were infected with VSV. Twenty-four hours later, and the viral load were measured by plaque assay **(F)**. Wild type or *Sting*^gt/gt^ peritoneal macrophages were treated with Dpo, 4 h later infected with HSV-1, and the mRNA level of ISG15 were measured by qPCR. **(G)** *Sting*^gt/gt^ C57BL/6 mice were treated with Dpo, 24 h later infected with HSV-1, and monitored daily for 15 days. **(A,B,D–F)** The data represent mean values ± SEM (*n* = 3); ^*^P < 0.05, ^**^P < 0.01 significant compared to control, Student's *t*-test. The data represent mean values ± SEM (*n* = 6 mice per group); **(C–G)** ^*^P < 0.05, significance compared to the control group, non-parametric Mann–Whitney analysis.

Innate immunity is considered to act as the first line of host defense and is critical for virus surveillance. Toll like receptor (TLR) is the first identified PRRs family in mammals that is able to recognize the pattern recognition proteins (PRPs) from different types of pathogens including parasite, fungi, bacteria and virus. All of the TLRs transduce danger signals through adaptor protein MyD88 (Häcker et al., 2000; Takeuchi et al., 2000a,b) except TLR3 that utilize TRIF (Yamamoto et al., 2002) as the adaptor. We first examined the possibility that Dpo was sensed by TLRs. However, the ISG15 induction by Dpo was intact in MyD88 or TRIF deficient macrophages (Figure 3A), so was the inhibition of viral replication (Figure 3B). Consistently Dpo also could protect TLR4 deficient mice from lethality after HSV-1 infection (Figure 3C). Thus, Dpo did not activate innate immune signaling through TLRs. RLR, including RIG-I and Mda5, is responsible for sensing the cytosolic RNA virus. MAVS is the adaptor protein downstream of RIG-I and Mda5 and critical for RLR signaling. MAVS transduces the signal from RLR to kinase TBK1, which in turn phosphorylate and activate IRF3. Activated IRF3 then translocate from cytoplasm to nucleus to initiate transcription of type I IFNs. We observed that Dpo was able to inhibit virus replication in RIG-I or MAVS deficient macrophages (Figures 3D,E), demonstrating that Dpo was not involved in RLR signaling. We then investigated the role of Dpo in cytosolic double strands DNA sensing pathway. STING is the key signal molecule of this pathway. We noted that Dpo enhanced immune responses were greatly attenuated in STING deficient cells during viral infection (Figure 3F). In line with this, Dpo treatment could not rescue STING deficient mice from death caused by viral infection (Figure 3G). Collectively these results demonstrated that Dpo modulated cytosolic double strands DNA sensing signaling in STING dependent manner.

### Dpo induces ISGs via IRFs/ELF4

**Figure 4:**
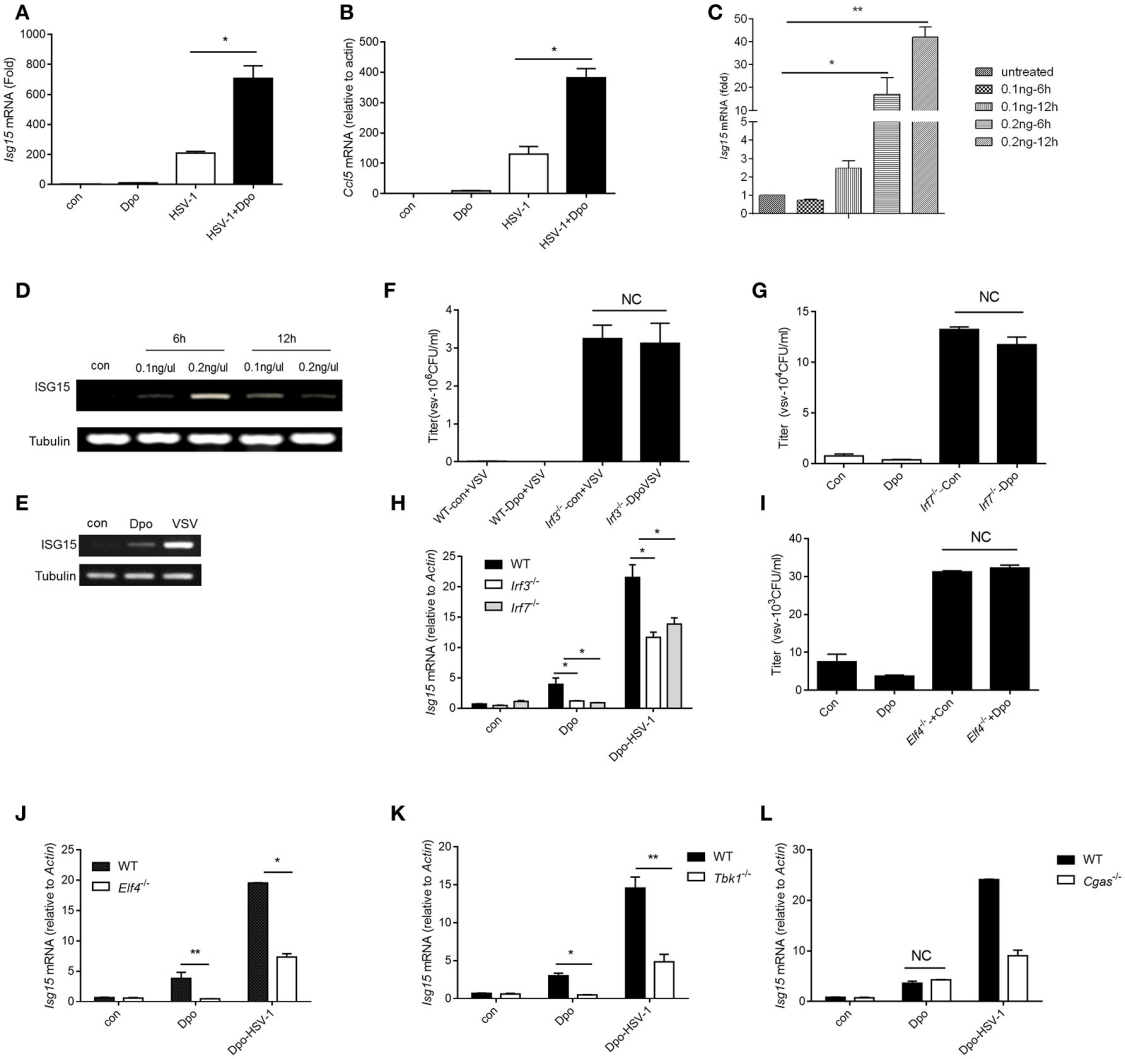
Dpo induces ISGs via IRFs/ELF4. **(A,B)** Bone marrow derived macrophages were treated with Dpo. Four hours later, these cells were infected with HSV-1. Twenty-four hours later, the mRNA level of ISG15 **(A)** and CCl5 **(B)** were measured by qPCR. **(C)** Bone marrow derived macrophages were treated with increasing amount of Dpo. Twenty-four hours later, the mRNA level of ISG15 was measured by qPCR. **(D,E)** DC2.4 cells and peritoneal macrophages were treated with Dpo or infected with VSV. Twenty-four hours later, the mRNA level of ISG15 were measured by RT-PCR. **(F–H)** WT, *Irf3*^−/−^ or *Irf7*^−/−^ iBMDM were treated with Dpo. Four hours later, these cells were infected with VSV. Twenty-four hours later, and the viral load were measured by plaque assay **(F,G)**, the mRNA level of ISG15 was measured by qPCR **(H)**. **(I,J)** WT or *Elf4*^−/−^ iBMDM were treated with Dpo. Four hours later, these cells were infected with HSV-1. Twenty-four hours later, and the viral load were measured by plaque assay **(I)**, the mRNA level of ISG15 was measured by qPCR **(J)**. **(A–C,F–J)** The data represent mean values ± SEM (*n* = 3); ^*^P < 0.05, ^**^P < 0.01 significant compared to control, Student's *t*-test. WT, *Tbk1*^−/−^**(K)** or *Cgas*^−/−^ **(L)** iBMDM were treated with Dpo. Four hours later, these cells were infected with HSV-1. Twenty-four hours later, the mRNA level of ISG15 was measured by qPCR.

We have found that Dpo was able to enhance the induction of ISGs by viral infection. We then checked the possibility whether Dpo could induce ISGs expression by itself in the non-infected cells. q-PCR analysis showed that Dpo was able to up-regulated the expression of CCl5 and ISG15 without virus challenging (Figures 4A–E). It is established that the robust induction of ISGs is directly mediated by innate transcription factors. We next assessed the role of known transcription factors in ISGs regulation after Dpo treatment. Dpo could not suppress virus replication in IRF3 or IRF7 deficient macrophages (Figures 4F,G). Consistently the induction of ISG15 by Dpo was attenuated in IRF3 or IRF7 deficient macrophages (Figure 4H), demonstrating that Dpo related anti-viral function is mediated by IRFs. ELF4 is an ETS domain containing transcription factor. Our previous study showed that it was critical for antiviral immunity. ELF4 cooperated with IRF3/IRF7 to initiate the transcription of antiviral genes. We thus evaluated the function of ELF4 in Dpo mediated immune responses. The plaque assay showed that Dpo inhibited the virus replication in wild type but not ELF4 deficient macrophages (Figure 4I). The induction of ISG15 was also impaired in ELF4 deficient cells (Figure 4J). CGAS is the cytosolic DNA sensor that synthetizes cGAMP to activate STING and TBK1. We found that TBK1 was indispensable for Dpo mediated ISG induction but cGAS was dispensable (Figures 4K,L). Therefore, Dpo regulates ISGs induction in a TBK1/IRF3/IRF7/ELF4 dependent way.

## Discussion

E. fischeriana root has been used to cure immune diseases in Chinese medicine for thousands years. The active compounds within it and the mechanism of how they modulate host immune system is largely unknown. There are approximate 60 compounds of E. fischeriana root isolated since the 1970s. EtOH and H_2_O extracts of E. fischeriana were found to inhibit the growth of Lewis lung carcinoma and ascetichepatoma in mice (Yang, 1993). Several studies on jolkinolide B (Uemura and Hirata, 1972) have been conducted in order to better understand the underlying mechanism of the antitumor activity of E. fischeriana. In addition, five extracts exhibited antibacterial activities against tuberculous bacillus and Prostratin was useful in the treatment of HIV. However, their function in innate immune pathway is unclear.

We here demonstrated that the E. fischeriana root compound Dpo enhanced antiviral innate immunity. The mechanism is different from prostratin mediated anti-HIV responses. The main mechanisms of HIV-1 inhibition effects by prostratin involved: (1) down-regulation of CD4 receptor expression as well as HIV-1 coreceptors, C-X-C chemokine receptor type 4 (CXCR4) and C-C chemokine receptor type 5 (CCR5; Kulkosky et al., 2001) and leading to protecting CD4+ T cells from HIV-1 infection. (2) up-regulation of activation receptor. (3) Leading to virus replication in latently infected cells. As a protein kinase C (PKC) agonist, prostratin activates PKC and cause phosphorylation of IκB kinase, which leads to NF-κB entering into the cell nucleus and promoting antiviral gene expression. This induction activity facilitate the eradication of viruses through elimination of their reservoirs. However, Dpo exerted its anti-viral function by activating innate immune signaling. STING is known as a type I IFNs stimulator localized on endoplasmic reticulum. Recent studies revealed its role as a sensor that recognized the secondary messager cGAMP (Cyclic GMP-AMP). Our *in vivo* and *in vitro* results showed that STING was essential for Dpo induced immune response. The detailed mechanism how Dpo activates STING signaling is still interesting to further explore. Similar to cGAMP, Dpo is a small molecule compound. It is possible that Dpo act as an agonist sensed by STING, and then activate downstream signaling.

ISGs are interferon stimulated genes that direct antiviral responses and immune homeostasis. The canonical induction of ISGs signaling are triggered by interferon through interferon receptors. Previous study had showed that viral infection was able to up-regulate the expression of ISGs directly through MAVS signaling without activation of IFN receptor signaling (Dixit et al., 2010). We here reported that Dpo initiated ISGs production in STING dependent but type I IFNs independent way. IRF3/7 and ELF4 are key transcription factors for type I IFN expression. Our previous study has shown that EICE (ETS–ISRE composition element) mediated the cooperation between IRFs and ELF4 in type I IFN transcription. IRF3/IRF7 and ELF4 are required for the induction of ISGs by Dpo, which should be bridged by EICE. Thus, our study uncover the role of an E. fischeriana root compound Dpo in innate immunity, and a STING/IRFs/ELF4 dependent pathway in ISGs regulation.

## Materials and methods

### Mice, cells, viruses, and reagents

*Sting*^gt/gt^ mice, which lack a functional STING protein, were a gift from R. Vance (University of California, Berkeley). The *Mavs*^−/−^ mice were from Jackson laboratory (Stock No: 008634) and the *Tlr4*^−/−^ mice were purchased from National Resource Center of Model Mice (NRCMM, Nanjing; N000192). All procedures followed the Peking University Guidelines for “Using Animals in Intramural Research” and were approved by the Animal Care and Use Committee of Peking University. iBMDM cells were a gift from Feng Shao (National Institute of Biological Sciences, Beijing). Elf4^−/−^, Irf3^−/−^, Irf7^−/−^, Myd88^−/−^, and Trif^−/−^ iBMDM were generated by Crispr-Cas9 system. HEK 293T (Human Embryonic Kidney 293 cells transformed by expression of the large T antigen from SV40), HeLa (Henrietta Lacks strain of cancer cells), Vero cell (normal African green monkey kidney epithelial cells), were cultured in Dulbecco's modified Eagle medium (DMEM). Bone marrow-derived macrophages (BMDMs), and macrophages was performed as previously described (Chen et al., 2011). Cells were cultured in 10 cm petri-dish at 37°C for 5 days. The medium for BMDCs is RPMI-1640 medium containing GM-CSF at 100 U/mL and TNF at 50 U/mL. VSV-GFP (Indiana strain) was gift from Dr. John Rose (Yale University) and HSV-1 was from Dr. Akiko Iwasaki (Yale University). LPS (lipopolysaccharides, L4391) was from Sigma. Lipofectamine 2000 (Invitrogen) was used for lipid transfection.

### *In vivo* Dpo treatment and virus infection

The C57BL/6 STING^−/−^, TLR4^−/−^ mice were breeding by ourselves. Age-and sex-matched C57BL/6 littermate were produced and used in all the experiments.

Seven-week-old mice were distribution into two groups: control group and experimental group. Before infection the mice with viruses, control group mice were treatment with PBS (200 μl/mouse), and experimental group mice with Dpo (10 μg/mouse) by intravenous injection. Twenty-four hours later, both group mice were infection with HSV-1 with 2 × 108 plaque-forming units (PFU) of viruses per mouse by intravenous. The survival state of the mice was monitored accordingly.

### Pseudo typed HIV-1 production and infection

Vesicular Stomatitis Virus Glycoprotein (VSV-G)-pseudotyped HIV or YU2-HIV were produced from 293T cells using the standard phosphate calcium transfection protocol. HEK293T cells were seeded at a density of 8 × 105 cells per well in 6-well plates. Twelve hours after plating, cells were transfected with VSV-G (0.07 μg) or YU2 (1.33 μg), pOPRIgagpol (1.5 μg), Ps-ReV (0.33 μg) and HIVec2GFP (0.66 μg), media was replaced 16 h after transfection and viruses were harvested 48 h later, filtered at 0.2 mm, and stored at −20°C in aliquots (After 10 min centrifugation at 14 krpm, virus-containing supernatant was transferred to a fresh tube, and stored at −20°C in aliquots). HEK293T (2 × 10^5^) and TZM-bl cells (1 × 10^5^) were plated on 24 well-plates 12 h before infection. Infection was performed by addition of 300 μL virus-containing supernatant. After a 12-h incubation, supernatants were removed and replaced with fresh media. Luciferase assay was measured 48 h after infection.

### Quantitative real-time PCR

Total RNA was isolated from cells using the RNeasy RNA extraction kit (Qiagen) and cDNA synthesis was performed using 1 μg of total RNA (iScriptcDNA Synthesis kit). Quantitative PCR was done with gene-specific primers and 6FAM-TAMRA (6-carboxyfluorescein–N,N,N′,N′-tetramethyl-6-carboxyrhodamine) probes or inventoried gene expression kits from Applied Biosystems [6FAM-MGB (6-carboxyfluorescein minor groove binder) probes]. The sequence of the primers were listed in the Supplementary Table 1.

### Plaque assay

For the HSV-1 plaque assays, cells (~1 × 10^5^) were first infected with virus. Twenty-four hours later, supernatants were collected and used to infect confluent Vero cells. One hour later, supernatants were removed and cells were washed with PBS and culture medium containing 2% (wt/vol) methylcellulose was overlaid for 60 h, cells were fixed for 30 min with 0.5% (vol/vol) glutaraldehyde and were stained with 1% (wt/vol) crystal violet dissolved in 70% ethanol. Plaques were counted and average counts were multiplied by the dilution factor to determine the viral titer as plaque-forming units per ml. The VSV plaque assay was performed as previously described (You et al., 2009).

### RNA seq

Macrophages were treated with Dpo or medium. We harvested these cell and control cells, and purified whole RNA by using RNeasy Mini Kit (Qiagen NO. 74104). The transcriptome library for sequencing was generated using VAHTSTM mRNA-seq v2 Library Prep Kit for Illumina® (Vazyme Biotech Co., Ltd, Nanjing, China) following the manufacturer's recommendations. After clustering, the libraries were sequenced on IlluminaHiseq X Ten platform using (2 × 150 bp) paired-end module. The raw images were transformed into raw reads by base calling using CASAVA (http://www.illumina.com/support/documentation.ilmn). Then, raw reads in a fastq format were first processed using in-house perl scripts. Clean reads were obtained by removing reads with adapters, reads in which unknown bases were more than 5% and low quality reads (the percentage of low quality bases was over 50% in a read, we defined the low quality base to be the base whose sequencing quality was no more than 10. At the same time, Q20, Q30, GC content of the clean data were calculated (Vazyme Biotech Co., Ltd, Nanjing, China). The original data of the RNA-seq was uploaded to the GEO DataSets (GEO Accession No. GSE104236).

### Statistical analysis

Statistical comparisons were performed with the mean ± standard error of the mean (SEM) for continuous variables. All data were statistically analyzed by unpaired 2 sample *t*-test with *p* < 0.05 indicative of statistical significance. All analyses were performed using GraphPad Prism 6.

## Author contributions

FY: Designed the study and analyzed the data and wrote or revised the paper. JC, TL, HD: Performed experiments. LY: Analyzed the data and provided expertise and contributed to animal work. SC: Provided expertise and contributed to RNAseq screening. WT, HS, BJ, GY: Provide technical help. LY: Provided expertise and contributed to experiment with viral infection.

### Conflict of interest statement

The authors declare that the research was conducted in the absence of any commercial or financial relationships that could be construed as a potential conflict of interest.
