# Supplementary figures and images for: E. fischeriana Root Compound Dpo Activates Antiviral Innate Immunity
Source: Front Cell Infect Microbiol. 2017 Oct 26;7:456. doi: 10.3389/fcimb.2017.00456 (PMC5662903; doi:10.3389/fcimb.2017.00456)

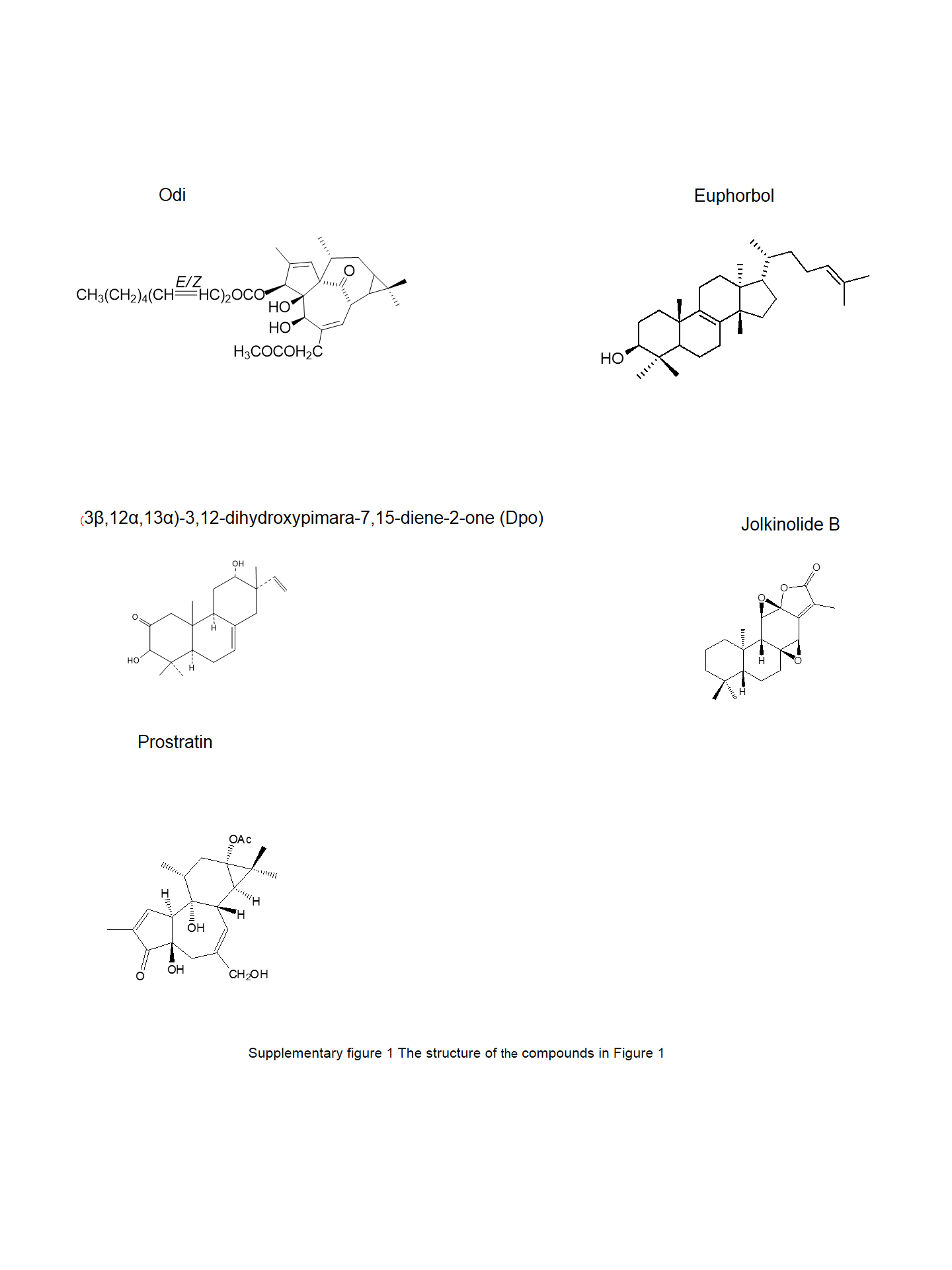

Supplement: Supplementary file 2 [file Image1.TIF]

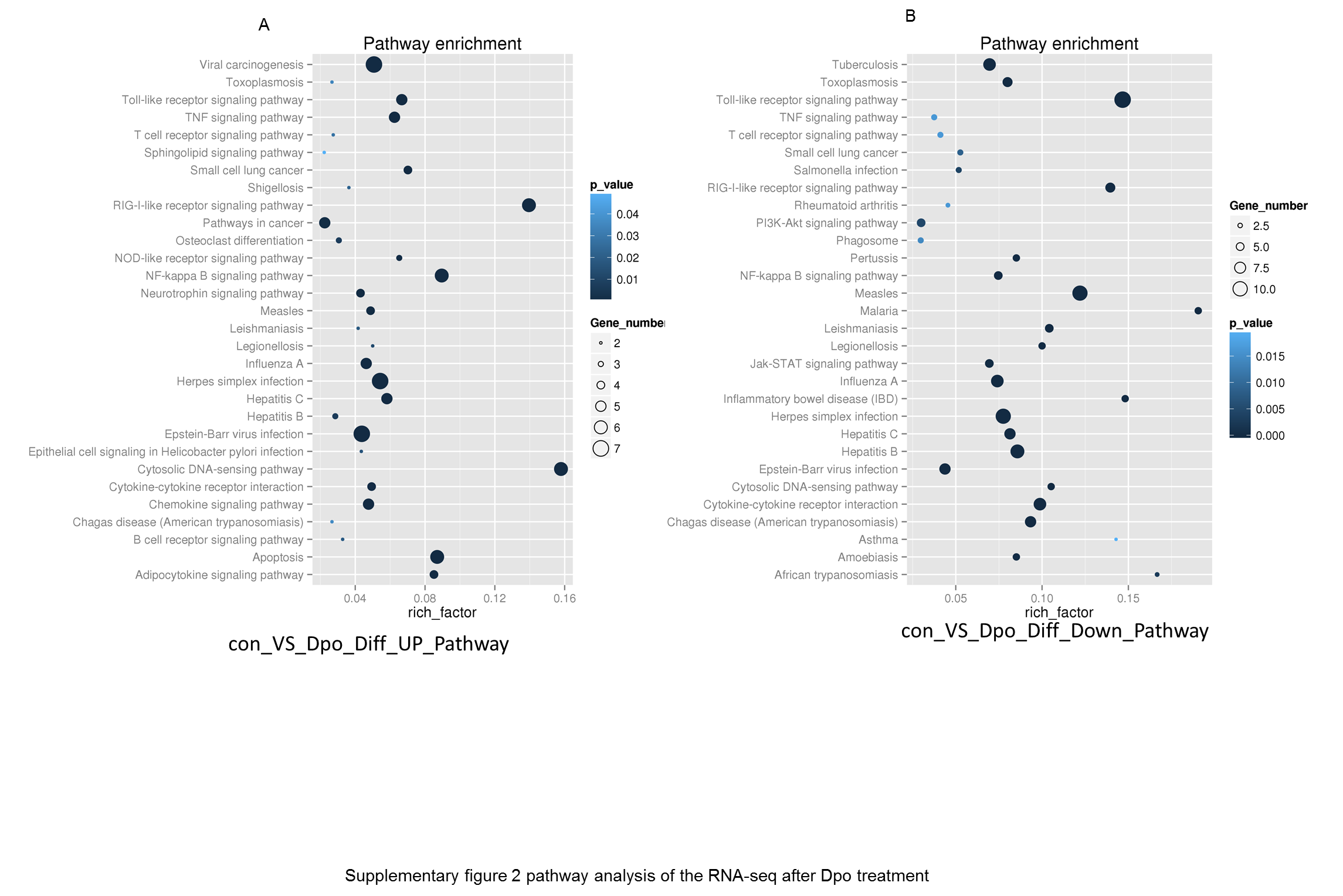

Supplement: Supplementary file 3 [file Image2.TIF]
